# New Frontiers in Breast Cancer Imaging: The Rise of AI

**DOI:** 10.3390/bioengineering11050451

**Published:** 2024-05-02

**Authors:** Stephanie B. Shamir, Arielle L. Sasson, Laurie R. Margolies, David S. Mendelson

**Affiliations:** Department of Diagnostic, Molecular and Interventional Radiology, The Icahn School of Medicine at Mount Sinai, 1 Gustave L. Levy Pl, New York, NY 10029, USA

**Keywords:** artificial intelligence, deep learning, mammography, ultrasound, MRI, breast cancer, CNN, risk stratification

## Abstract

Artificial intelligence (AI) has been implemented in multiple fields of medicine to assist in the diagnosis and treatment of patients. AI implementation in radiology, more specifically for breast imaging, has advanced considerably. Breast cancer is one of the most important causes of cancer mortality among women, and there has been increased attention towards creating more efficacious methods for breast cancer detection utilizing AI to improve radiologist accuracy and efficiency to meet the increasing demand of our patients. AI can be applied to imaging studies to improve image quality, increase interpretation accuracy, and improve time efficiency and cost efficiency. AI applied to mammography, ultrasound, and MRI allows for improved cancer detection and diagnosis while decreasing intra- and interobserver variability. The synergistic effect between a radiologist and AI has the potential to improve patient care in underserved populations with the intention of providing quality and equitable care for all. Additionally, AI has allowed for improved risk stratification. Further, AI application can have treatment implications as well by identifying upstage risk of ductal carcinoma in situ (DCIS) to invasive carcinoma and by better predicting individualized patient response to neoadjuvant chemotherapy. AI has potential for advancement in pre-operative 3-dimensional models of the breast as well as improved viability of reconstructive grafts.

## 1. Introduction

Breast imaging is an advanced subspecialty in the domain of radiology. It has benefited from rapid advances in imaging technology. This manuscript will describe the advances that artificial intelligence (AI) brings to this already well-established domain. We hope that we have provided enough context regarding the complex diseases and technologies relevant to breast imaging, so that the reader with only moderate knowledge of these may still understand the AI revolution in this domain.

Breast cancer is the most frequently diagnosed malignancy and one of the most important causes of cancer mortality among women [1]. Breast cancer accounts for 12.5% of all new annual cancer cases worldwide [2]. Breast cancer survival rates are based on several factors, including the stage of malignancy, with 3% of women potentially dying from breast cancer in their lifetime. Given its prevalence and the ever-growing oncologic needs of cancer patients [3], there has been a movement towards creating more efficacious methods for breast cancer detection, including the development of state-of-the-art imaging technologies. In the recent past, this has involved the development of digital breast tomosynthesis (DBT) and multiparametric magnetic resonance imaging (MRI) [1], but now there has also been increased emphasis on using AI to improve radiologist accuracy and efficiency [3]. Breast cancer detection has several limitations, such as the growing demand for scans that obviate more time for interpretation (such as CT and MRI) with the persistent scarcity of radiologists available to read these imaging studies, the variation among clinician interpretation, and the fact that certain scans require specialized facilities and are quite expensive [4]. Amongst other benefits, AI can be used to improve image quality, increase interpretation accuracy, and improve time efficiency and cost efficiency [5].

AI is a vast, rapidly evolving, field encompassing multiple different technologies and applications to solve difficulties that typically necessitate human intelligence [5,6]. AI takes advantage of computer-based algorithms to perform these tasks. AI technologies have greatly improved in recent years, with a transition from machine learning to deep learning and now to transformer models that can combine information from various modalities as inputs. Convolutional neural networks (CNNs) are widely used in deep learning, as they can extract spatial and contextual information from images through multiple layers. Transfer learning is an important method that allows the transfer of learned features to new tasks with limited labeled data, reducing the need for extensive training [4]. Utilizing AI in the realm of medicine, and specifically in the radiographic assessment of malignancy, offers many benefits to clinicians [7]. Through its ability to discern complicated image patterns, AI allows for the automation of image interpretation and the diagnosis of diseases, including breast cancer [1,7].

AI can also assist in the nonquantitative assessment of cancer imaging, such as prediction of tumor genotype, the impact of disease, preoperative neoadjuvant chemotherapy response, and treatment-related effects on adjacent organs [7,8]. Further, machine learning can be utilized to predict the upstaging risk of DCIS to IDC, utilizing mammography and MRI, and thus identify significantly more women eligible for the Comparison of Operative versus Monitoring and Endocrine Therapy (COMET) active surveillance trial [9,10,11].

Mammographic AI can be used as a prognostic tool, utilizing automated breast density and individual clinical factors to predict breast cancer risk [12,13]. AI-assisted systems have also refined the performance of imaging modalities in the automatic identification and differential diagnosis of breast lesions [14]. Furthermore, when a breast lesion has been detected, AI support can prove beneficial in the preoperative period [15].

AI techniques can be used to examine breast factors such as symmetry, volume, and shape during surgical planning. Preoperative imaging studies can also characterize the vascular supply of the breast, and with this information, AI algorithms can help determine which reconstructive techniques are the most reliable during breast surgery [16].

Our paper provides an exhaustive description of the multimodal AI technology available, including mammography, ultrasound, and MRI. It is the most extensive review we have seen related exclusively to the implementation of AI in breast imaging.

## 2. Imaging Modalities and Their Advances

This section will undergo a comprehensive review of some of the more frequently utilized imaging modalities in the field of breast imaging (Table 1). AI techniques have been shown to improve breast cancer imaging in a variety of ways, including through initial cancer detection as well as the prognostication and risk stratification of breast cancer. We will discuss how the implementation of AI has been employed to more effectively operate these imaging technologies in these avenues and more (Table 2).

### 2.1. Mammography

#### 2.1.1. Technique

Screening mammography has the most machine learning and deep learning models available [17]. While diagnostic algorithms receive much attention, there are many other ways in which AI can be applied in healthcare. AI can be used to improve the quality of mammograms, such as in some systems that provide real-time feedback to mammography technologists regarding positioning and quality control metrics. AI implemented in a low-resource clinical setting, for example, provided approximately 20-point improvements in posterior breast tissue inclusion in screening mammograms over a 10-week period [18]. Beyond imaging quality improvements, mammographic AI has vast clinical potential.

#### 2.1.2. Cancer Detection

The utilization of non-human methods to assist with mammographic reading is not a new one. Computer-aided detection (CAD) was initially thought to be used as a “second pair of eyes” in place of two radiologists reading a study, otherwise referred to as, double reading [19,20]. While CAD can reduce the interpretation time of DBT by 29.2%, it is associated with a significant increase in recall rates. A 2011 study that used data from the United Kingdom CADET II study found that when assessing the cost-effectiveness, measured in terms of cost per cancer detected, of one radiologist reading with CAD versus two radiologists reading, CAD is unlikely to be cost-effective secondary to the added cost from higher recall rates [19,20]. Further, a study performed in the United States demonstrated that CAD applied to film-screen actually reduced specificity and did not improve cancer detection rates [21]. A later study applied to digital mammography reaffirmed these results and demonstrated that CAD did not improve screening accuracy [22]. Automated methods utilizing the stratus method and CAD mammographic features (density, masses, and microcalcifications) have advanced since CAD was first implemented [23]. However, AI has improved and expanded from CAD.

**Figure 1:**
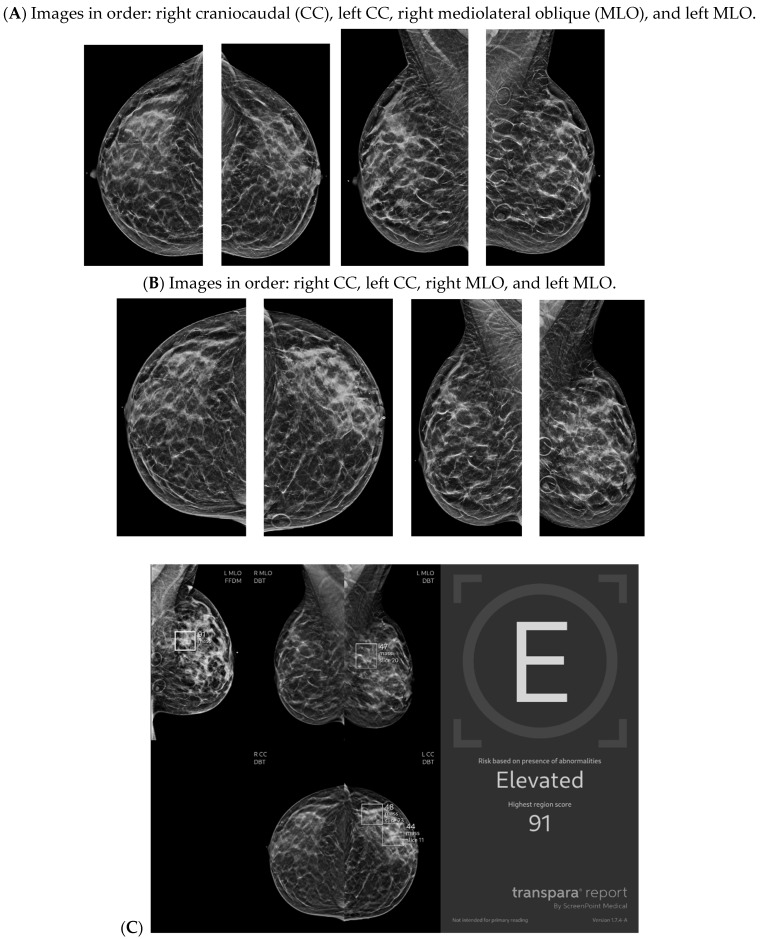

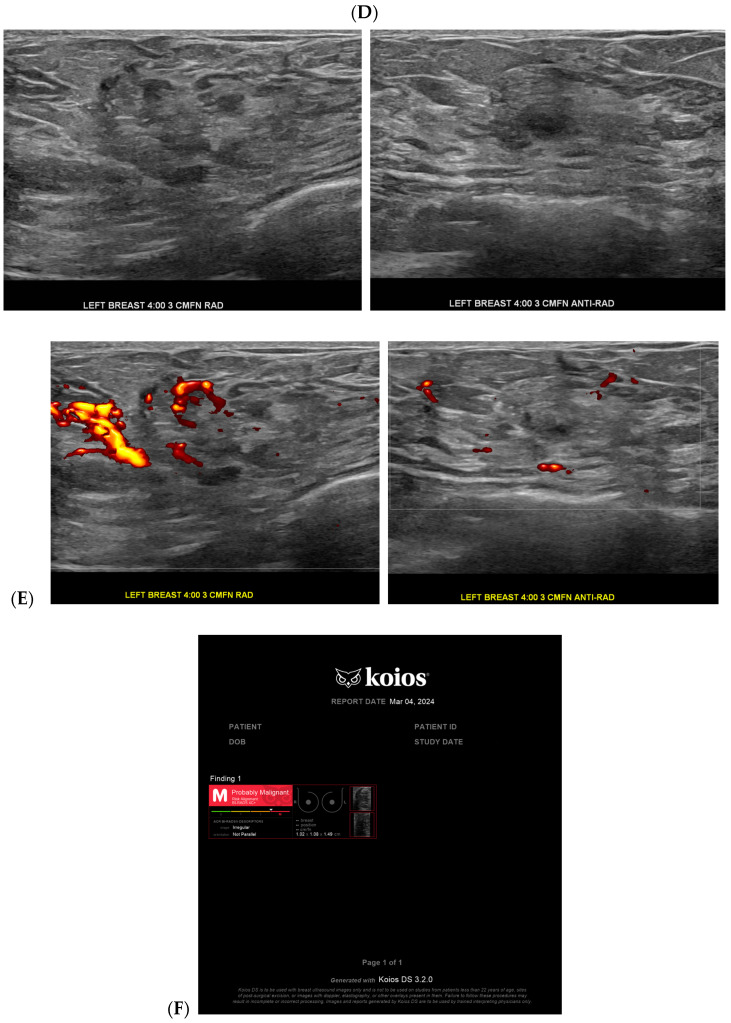
Developing asymmetry detected by artificial intelligence (AI): Between the baseline screening mammogram (**A**) and the follow-up screening mammogram 17 months later (**B**), there has been a very subtle development of left breast asymmetry that is difficult to perceive with the naked eye. However, the AI program Transpara highlighted potential regions of interest (**C**) for the radiologist to query for additional mammographic and sonographic imaging. On further diagnostic imaging, the subtle asymmetry corresponds to a hypoechoic mass at left 4:00, 3 cm FN (**D**) with hypervascularity (**E**). AI program Koios correctly recognized the mass as “Probably Malignant”, and this area returned as a biopsy-proven invasive malignancy with lymphangitic spread (**F**). Images obtained from the Icahn School of Medicine at Mount Sinai.

**Figure 2:**
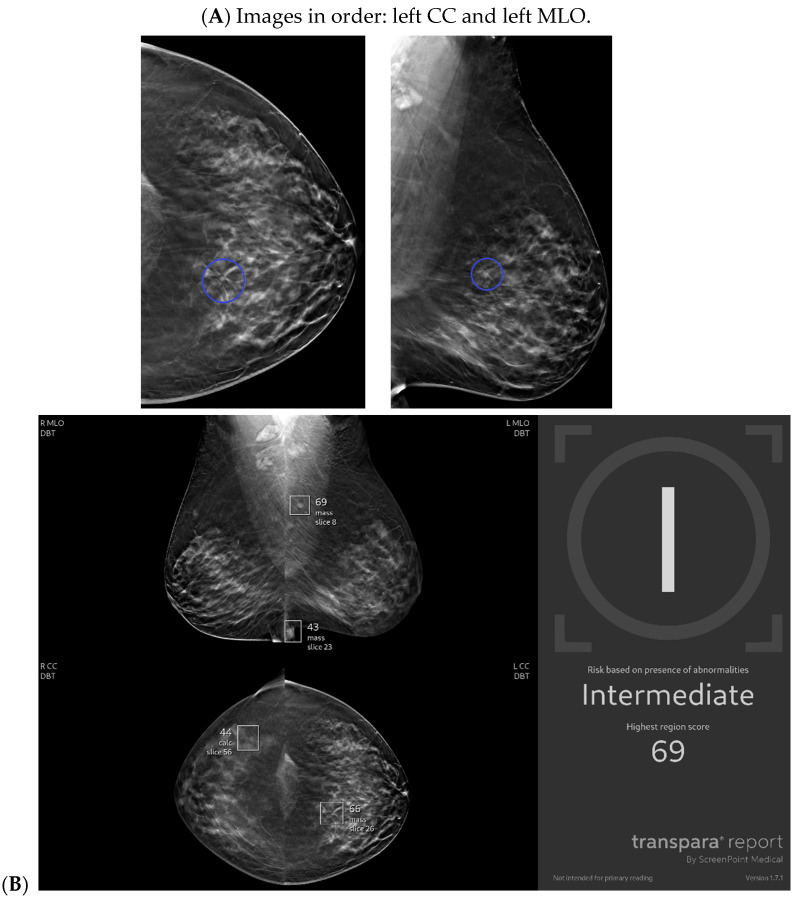
New architectural distortion detected by artificial intelligence (AI): A patient in her 50s’ screening mammogram revealed a new area of architectural distortion (circle) in the inner central region of the left breast (**A**). The AI program Transpara highlighted potential regions of interest, including this suspicious area of architectural distortion on the left breast on the corresponding left CC view; however, AI also highlighted benign areas that were arbitrated out by the radiologist (**B**). There was no sonographic correlate, so a stereotactic biopsy of this area of architectural distortion was then biopsied under guidance. Pathology yielded invasive lobular carcinoma. Images obtained from the Icahn School of Medicine at Mount Sinai.

With the widespread implementation of DBT, which increases mammographic cancer detection sensitivity, there are larger volumes of images. This may ultimately increase the risk of reduced accuracy, perhaps due to reader fatigue and increased reading time [24]. There are numerous tomosynthesis AI products available, and at our institution we have implemented the use of Transpara, which is a deep learning-based AI system that uses deep CNN to help improve early-stage cancer detection and sensitivity (at similar specificity), while reducing reading time [24,25]. We view tomosynthesis AI as a valued asset to our clinical practice, as the software highlights potential areas of concern that require additional attention (Figure 1 and Figure 2). However, some areas flagged as concerning are often classically considered benign, such as stable post-lumpectomy sites, stable asymmetries and calcifications, or previously biopsied benign findings. That being said, tomosynthesis AI has been found to have a synergistic effect on cancer detection rate (CDR) when utilized by the radiologist. A study found that radiologist-only CDR was 67.3% and AI-only CDR was 72.7%, but when the radiologist and AI software were used together, the CDR increased to 83.6% [17,25,26]. Lunit INSIGHT MMG, Seoul, South Korea was the diagnostic support software used in this particular study [26].

An additional study found that a radiologist’s use of AI had a synergistic effect on sensitivity and specificity; the German national breast cancer screening program found that AI alone had lower sensitivity and specificity, by 2.6% and 2.0%, respectively, than a radiologist; however, the combination of AI and a radiologist increased the sensitivity and specificity by 2.6% and 1.0%, respectively, when compared with a radiologist alone [27].

Some studies have demonstrated situations in which AI can be superior to that of a reading radiologist. For instance, a study found that AI was able to detect interval cancers that were not found by radiologists [28]. Further, at times, a radiologist actually “arbitrated out” interval cancers detected by AI [28]. This same study did, however, find that radiologist arbitration was also able to correct AI false-positives [28]. AI has been implemented on mammograms that have been deemed benign by the radiologist, and AI was used to extract mammographic features such as density, masses, and asymmetries to predict 30% of stage 2 and higher breast cancers in 6% of high risk women [29].

AI’s ability to detect cancer certainly gives vast clinical benefits to radiologists. Though mammographic AI cannot be used as a stand-alone reader or diagnostician at this time, the synergistic effect of a radiologist utilizing AI is certainly of importance. Additionally, AI’s detection capabilities also have the potential to triage screening workloads, as has been found for 2D mammography [30,31]. Breast imagers often have dozens to hundreds of screening mammograms in a queue to be interpreted. The triage by AI software to identify the exams most likely to reveal cancer can prioritize those patients to get quick attention.

#### 2.1.3. Prognostic Factors

AI techniques can act as a prognostication tool, with several examples already widely recognized in the realm of breast cancer imaging. Breast density can be estimated by the amount and distribution of breast fibroglandular tissue visualized on mammographic images and delegated into four different Breast Imaging-Reporting and Data System (BI-RADS) categories ranging from almost entirely fatty to extremely dense [6]. Not only does increased breast density lower the sensitivity of mammography, but increased breast density is associated with an elevated risk of developing breast cancer [12]. Oftentimes, there can be variability amongst radiologists in breast density reporting, which calls attention to the usefulness of computer-based evaluation in the standardization of breast density quantification [6,32]. Mammographic density can be determined using thresholding techniques or using fully automated methods [12]. Several FDA-approved models for breast density reporting currently exist, with studies revealing that there may be benefits to automated density reporting in cancer risk stratification [12,32]. Other mammography-based risk models can combine mammographic features with individual clinical factors to predict the risk of developing breast cancer within the next five years [13].

The reliability of automated breast density has been questioned as differences in positioning, compression, and technical parameters may affect measurements [33]. However, automated breast density has demonstrated low variability in repeated breast density measurements [33], and an additional study demonstrated reasonable agreement for breast volume for some mammographic AI methods [32]. Interestingly, one study demonstrated that visual assessment of breast density using a visual analogue scale (VAS) was the strongest predictor of breast cancer risk compared to other automated methods [12]. One must keep in mind that we are truly in the early stages of AI in healthcare, and diagnostic algorithms may certainly have room for improvement as the science of “deep learning” and “transformer models” evolves.

Measurement of fibroglandular tissue is another potential predictor of risk, as breast cancer is known to originate in fibroglandular tissue [32]. A study found that an AI program is reliable in estimating the local glandular tissue distribution and can be used for its assessment and follow-up [34]. However, divergences can arise with differences in breast compression [34], and there is less agreement for median fibroglandular tissue volume between programs [32].

#### 2.1.4. Risk Stratification

DCIS is a proliferation of malignant epithelial cells that are bound by the mammary duct basement membrane [35]. Due to the possible differences in management of DCIS from invasive carcinoma, it is critical for the correct detection of invasive cancer upon biopsy. CNN and radiomics applied to mammographic images were able to distinguish between pure DCIS and DCIS with invasion with high specificity, which has potential use in selecting patients for DCIS observation trials, such as the COMET trial or LORIS [10,36].

A time-modulated long short-term memory network based on deep learning or radiomics has been utilized to identify the likelihood of a breast lesion representing malignancy, both in a breast already affected by cancer and the contralateral breast [37].

Deep learning modeling using CNN to evaluate previously normal mammograms has been found to be predictive of short-term breast cancer risk [38]. AI deep-learning risk models utilizing data from mammograms also performed significantly better in estimating the short-term risk of developing breast cancer compared to traditional models that typically factor in personal and family history to determine the 10-year risk and lifetime risk of developing breast cancer, specifically the Tyrer–Cuzick model [39,40,41]. Further, a short-term risk AI model utilizing the Multiple Instance Learning model performed better than the VAS, as read by two experienced radiologists [40].

### 2.2. Ultrasound

#### 2.2.1. Cancer Detection and Diagnosis

**Figure 3:**
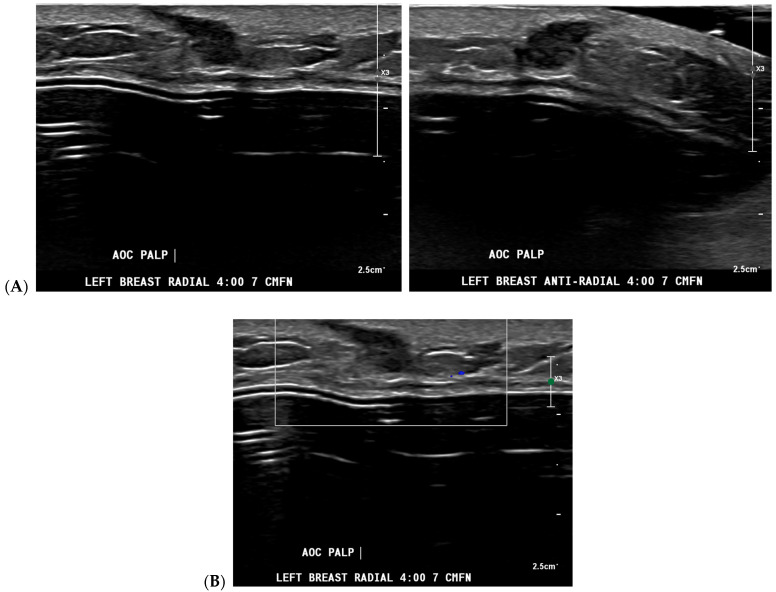

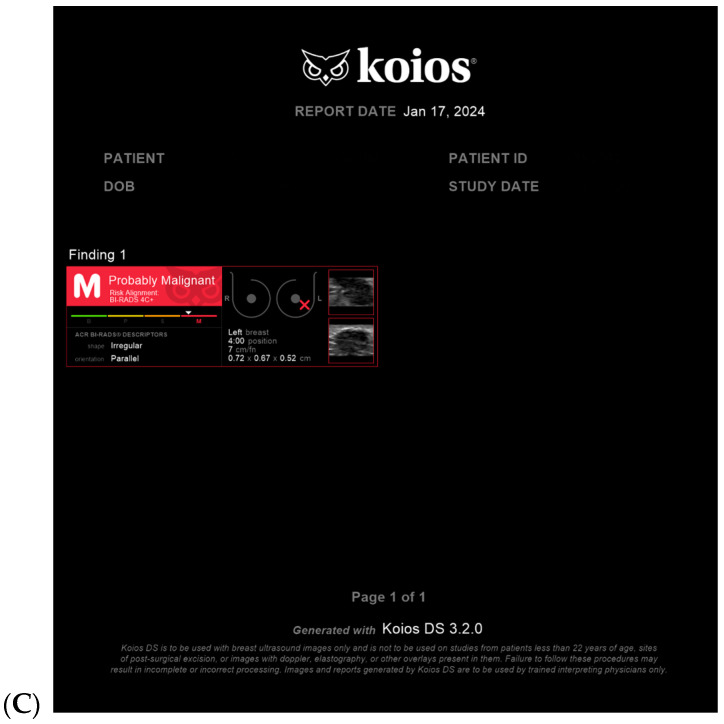
A new cancer diagnosis appropriately classified as “malignant” by artificial intelligence (AI): This patient in her 40s with a history of left breast carcinoma diagnosed 1 year prior, status post-left mastectomy with chemotherapy and hormonal therapy, presented with a palpable abnormality in the superficial lower outer left breast. No new or suspicious findings were seen on the patient’s diagnostic mammogram. Correlating with the patient’s concern about a palpable lump, diagnostic ultrasound revealed an irregularly shaped, hypoechoic mass with angular margins that are non-parallel (**A**), and Doppler shows no vascularity (**B**). The AI program Koios recognized this mass as “Probably Malignant” (**C**). This was returned as biopsy-proven invasive ductal carcinoma. Images obtained from the Icahn School of Medicine at Mount Sinai.

**Figure 4:**
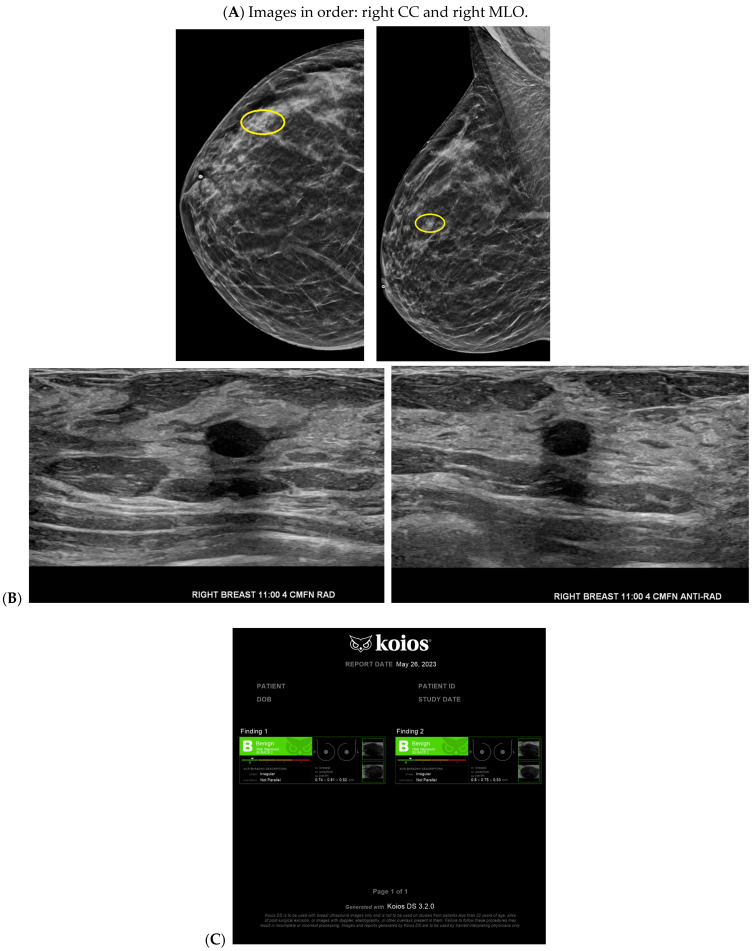
A benign finding appropriately classified as benign by artificial intelligence (AI): The patient initially presented for a bilateral screening mammogram and a bilateral screening breast ultrasound. A mammogram revealed benign dystrophic calcifications in the upper outer quadrant of the right breast (**A**). Correlating with findings on the mammogram, ultrasound revealed a complicated cyst showing posterior acoustic shadowing consistent with fat necrosis (**B**). The AI program Koios recognized this mass as “Benign” (**C**). Images obtained from the Icahn School of Medicine at Mount Sinai.

Cancer detection systems typically involve neural networks, machine learning, or deep learning developed from training models to recognize patterns, while diagnostic systems use an additional algorithm to classify [17]. Several CNN models have been developed to correlate ultrasound imaging features of a lesion with the four-classification breast cancer molecular subtypes [42]. Our institution uses Koios, which utilizes machine learning and AI to generate the probability of malignancy of a breast finding by evaluating a region of interest (ROI) selected by a radiologist [3,5]. Ultrasound AI has been found to reduce intra- and interobserver variability and to improve accurate BI-RADS classification of sonographic breast findings [3,5]. Ultrasound AI can also increase CDR [5] and reduce the number of unnecessary biopsies [5,43,44] (Figure 1, Figure 3, and Figure 4).

Various ultrasound AI applications exist; some rely on hand-held US, and others utilize automatic breast ultrasound for lesion classification using qualitative and/or quantitative classification of the relative probability of malignancy for user-selected or software-selected soft tissue lesions [8]. Ultrasound AI has been found to be useful in identifying challenging diagnoses. For instance, triple-negative breast cancer (TNBC), a biologically aggressive subtype of breast cancer, can be challenging to identify on US owing to its often relatively benign sonographic features, yet a recent study has demonstrated that ultrasound AI accurately recommends biopsies for 96% to 97% of TNBCs and even an accurate biopsy for six TNBCs initially misclassified by radiologists as benign or probably benign [5]. A study found that a deep learning-based CAD was more accurate at diagnosing benign entities than a radiologist and a resident and was equally accurate at diagnosing malignant entities [44].

From our personal experience, ultrasound AI is a welcome addition as it functions to help narrow the classification of an ambiguous lesion for sonographic findings that do not have a clear-cut BI-RADS classification, which can ultimately reduce deliberation time for findings that seem to be in between classifications.

There are challenges that arise with ultrasound AI. Limitations of ultrasound AI tend to involve its reliance upon operator-dependent factors, including the quality of static US images (related to equipment quality and operator-related factors) obtained and the ROIs selected for a lesion by the radiologist, which may introduce variability [5,8]. Additionally, AI does not account for data beyond 2D data that improves diagnostic accuracy, such as cine loops, elastography [45], and color doppler [5,8]. Further AI does not currently take into account other lesions within the breast when assessing a finding, and thus findings such as multiple bilateral, circumscribed oval masses, which are statistically benign, are not factored in, and instead each mass is assessed in isolation [8,46]. This is also relevant to masses that have demonstrated two, or greater, year stability and are thus known to be considered statistically benign to a radiologist but not factored into current AI algorithms. Lesion selection is an important part of our training to use Koios. That is, not all findings need to be evaluated, as no decision support is needed. Despite these potential limitations, US AI has demonstrated clinical utility with the added benefit of providing improved access in low-resource regions [8,46].

#### 2.2.2. Prognostic Factors

US features of TNBC have been found to be correlative with the expressions of mRNA and thus predictive of the risk of recurrence [47]. US-based radiomics analysis demonstrated a significant association between radiomic nomogram and disease-free survival and thus has the potential to be utilized for risk stratification [48].

#### 2.2.3. Surgical Planning

Ultrasound can be utilized to assess the vascular supply of the breast to determine the plausibility of reconstructive techniques [15].

### 2.3. MRI

#### 2.3.1. Technique

**Figure 5:**
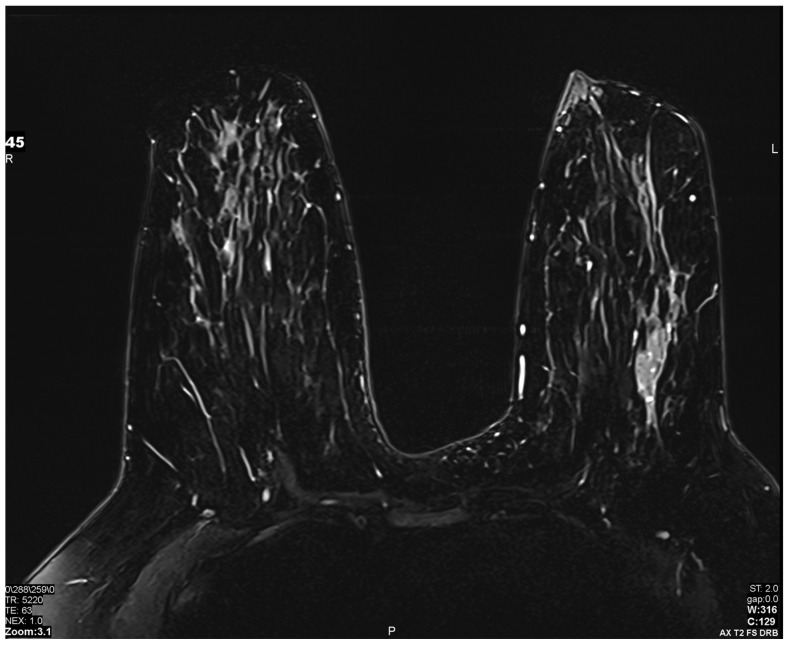
Artificial intelligence (AI) sequence utilized to accelerate image acquisition time: A deep resolve boost (DRB) AI sequence was utilized to increase the signal-to-noise ratio by artificially filling k-space, allowing for accelerated image acquisition. Images obtained from the Icahn School of Medicine at Mount Sinai.

Historically, MRI examinations may have required numerous imaging sequences over a prolonged period of time, all of which required the patient to remain still. AI techniques have been introduced to speed up signal processing and reduce image noise, resulting in quicker and equally accurate exams [49] (Figure 5). Additional techniques, such as synthesized MRI images, allow for a reduced need for contrast agents in producing images [50]. This technology has been increasingly made available in commercial MRI installations.

#### 2.3.2. Cancer Detection

MRI offers the highest sensitivity and specificity of all available breast imaging methods [51]. MRI provides large datasets, making it a suitable imaging study for the application of artificial intelligence [52]. MR images may encompass hidden information that might not be discernible by human evaluation but can be extracted using machine learning methods [53]. With these data, AI can be critical in the detection of lesions suspicious for breast cancer.

#### 2.3.3. Cancer Diagnosis–Lesion Characterization

A study utilizing computer-aided diagnosis software demonstrated notable diagnostic accuracy; more specifically, the average area under the curve (AUC) was higher when a radiologist utilized AI software (0.76) compared with a radiologist interpretation alone (0.71) [51]. Another study utilizing MRI machine-based learning to assess radiomic features extracted from contrast-enhanced T1-weighted and T2-weighted images has been found to assist in the diagnosis of contralateral BIRADS-4 lesions in women with breast cancer [54].

#### 2.3.4. Prognostic Factors

Background parenchymal enhancement (BPE) is a controversial topic, though studies have shown its potential use for breast cancer risk stratification [55,56,57,58,59,60], breast cancer hormonal receptor status [61,62,63], and cancer treatment response [64,65]. Thus, the ability to quantify BPE automatically has potential importance. Quantitative assessments of BPE have been developed; some use fully automated quantitative segmentation methods [64,66,67], while others use segmented semi-automatic methods [68]. The AI methods currently available offer a volumetric or qualitative computation of the fibroglandular tissue enhancement [69,70,71]. Studies have demonstrated that MRI breast images can be successfully organized by their background parenchyma enhancement through the application of a CNN and that this neural network is as accurate as a skilled radiologist [72]. A study testing breast MRI AI calculated a median AUC to be 0.80 for prognostic imaging and 0.85 for neoadjuvant therapy response.

#### 2.3.5. Risk Stratification

A core needle biopsy is oftentimes enough for a pathologist to make a DCIS diagnosis; however, the small tumor volume collected through a core needle biopsy may inadvertently omit neighboring invasive carcinoma [11]. Patients with a recent diagnosis of DCIS or invasive carcinoma regularly undergo preoperative breast MRI to evaluate the extent of the disease [73,74]. Some recent studies have revealed that semiautomatically assessed MRI features may play an important function in predicting patients with a preoperative diagnosis of DCIS who are also at an elevated risk for having a concomitant invasive carcinoma [11]. This MRI feature may assist clinicians in better tailoring the treatment plan for patients with underestimated breast cancer. One major advantage of utilizing this feature of MRI to predict DCIS upstaging is that the algorithmic assessment of lesions allows for a more standardized evaluation of tumor characteristics, as opposed to the subjective nature of a radiologist’s interpretation.

### 2.4. Other Relevant AI

#### Surgical Planning

Three-dimensional printing utilizes semi-automated techniques to produce models that can be used to evaluate treatment options, and assist with determining which treatment methods can achieve tumor-free margins with satisfactory cosmesis [75,76]. It also allows for optimized patient autonomy by granting patients the ability to visualize the extent of breast cancer and thus provide a better understanding of potential surgical treatment [76]. Three-dimensional printing can also be used to assist with breast reconstruction involving flap reconstruction by assisting with dissection of intramuscular perforator vessels, thus improving perfusion of flap reconstructions, and can help with breast radiation planning by creating customized brachytherapy templates [77]. AI can be utilized to enhance the production of 3D models and optimize the automation process, thus reducing the time required to create models, though it is not necessarily specifically available for breast imaging [15,76,78].

Prior to mastectomy reconstruction utilizing the deep inferior epigastric perforator (DIEP) flap technique, a pre-operative contrast-enhanced CT or contrast-enhanced MRI is usually performed in order to identify adequate perforators to ensure adequate perfusion post-operatively. A study utilized an AI technique applied to pre-operative angiographic CT scans and was found to be able to key perforators in greater than 97% of cases for DIEP flap reconstruction [15,79].

Though not directly imaging-related, a deep learning-based prediction model-based on patient specific factors can help anticipate post-operative reconstruction complications [80].

## 3. Discussion

AI has proven itself to provide meaningful clinical assistance to radiologists caring for patients’ breast health by utilizing multiple imaging modalities for cancer detection, diagnosis, prediction of prognosis, risk stratification, and surgical planning. Further, AI has the potential to become invaluable in the future for triaging patients in low-resource clinical settings, both abroad and nationally, in locations where there is a scarcity of subspecialized breast radiologists [5,8].

As of June 2023, there were more than 20 FDA-approved AI applications for breast imaging [6]. One study discovered that when comparing various different AI models applied to mammography, 10 of the 12 evaluated had greater than 90% accuracy [17,81]. It follows that selecting the appropriate AI vendor to purchase is a critical step in the clinical implementation of AI [82] and includes significant effort on the part of the stakeholders to evaluate the different available options on the market. Several practical frameworks can be utilized to evaluate AI products, such as the evaluating commercial AI solutions in radiology (ECLAIR) guidelines, which can recommend particular factors to consider during this selection process [83]. Factors to consider when evaluating an AI product include its relevance, the validation process, and how it can best be integrated into present clinical workflows [82].

Despite the variety of AI-based applications, there are still important barriers to the implementation of AI in breast practices. These may include significant AI program costs, inconsistent performance, and IT requirements [6]. Another barrier to clinical adoption could be the lack of radiologist, patient, and referring provider acceptance and trust in AI-based algorithms [82]. Radiologists have expressed concerns about the possible substandard performance of AI products, in addition to a possible reduction in productivity and reimbursement with the use of AI [84]. There are significant concerns regarding the possibility of bias in AI algorithms. AI algorithms are developed from large samples of training data. Sometimes these datasets are not truly representative of a diverse population and may not ultimately serve all racial, ethnic, and select socioeconomic groups well. Bias based on sex is an additional concern. Additionally, prior patient surveys have noted concerns over the possible future implementation of AI for standalone interpretation of breast imaging studies [85].

Beyond being utilized for the detection and diagnosis of breast cancer, mammography can also offer insight into a patient’s cardiac health through the demonstration of arterial calcifications. Automated AI detection algorithms for these calcifications are receiving more attention as potential means for highlighting patients at increased cardiac risk, which is especially important for women who are oftentimes not diagnosed with cardiac conditions in a timely fashion [86,87].

Despite hesitance by some radiologists to implement AI, radiologists that do utilize AI may be prone to another type of bias—“automation bias.” This bias is a tendency to favor machine-generated decisions over human intelligence [8]. For instance, a study found that radiologist performance in reading mammograms was weakened when receiving incorrect input from AI, and this was found to be especially true for less experienced radiologists [88]. It is important to be mindful of this bias when implementing AI so that there is a desired synergistic effect rather than reduced performance.

## 4. Conclusions

The current studies on AI implementation in mammography, US, and MRI demonstrate that, though AI is not currently accurate enough to make diagnoses alone, it has vast potential to supplement a radiologist [17]. Studies have shown that radiologist usage of AI has the ability to increase CDR [17,26], increase sensitivity and specificity [27], decrease false-negative diagnoses [26], improve accuracy [3,5], and improve efficiency [3,5], and reduce the number of unnecessary biopsies [5,43,44]. This can ultimately improve patient care.

## 5. Future Directions

Currently, much of AI functions in a vacuum without knowledge of patient symptomatology or findings on other imaging modalities [5]. Further, some AI does not currently take into account other lesions within the breast when assessing a finding [8,46]. Additionally, much current breast AI does not currently consider the stability of a finding, which is an important finding that radiologists rely upon to establish statistical benignity when a finding is stable for 2 years or more [89]. Future steps in AI can address these elements by closing the gap between providing diagnoses based solely on the input from a current breast focused study and taking into account a more holistic approach to the patient. These future models may factor in the patient’s medical and surgical history and other imaging findings from the same study or previous studies. More advanced techniques may consider findings from other modalities before coming to a final diagnosis. Additionally, related to ultrasound, optoacoustic breast imaging is an emerging field in which differences in thermoelastic expansion are utilized to help distinguish benign from malignant findings [90], and while this field evolves, possible implementation into AI algorithms may prove beneficial.

AI may better assist future surgeons with pre-operative planning as well as intraoperative imaging with augmented reality. Preoperatively, AI can be utilized to enhance the speed of 3D model production [15,76,78]. More rapid production of 3D models may help implement utilization and potentially provide useful input in surgical planning by guiding surgeons on where to place their scalpels. Further, virtual and augmented reality may be implemented in the future for breast surgery, as it has been for other specialties, most notably orthopedic surgery, and perhaps allow for the ability of a surgeon to wear a virtual headset to visualize relevant pre-operative imaging in real time while the patient is already on the operating table.

## Figures and Tables

**Table 1:** Imaging modalities and their advances, with associated reference numbers.

Topic	References
**Mammography**	
Technique	[17,18]
Cancer detection	[17,19,20,21,22,23,24,25,26,27,28,29,30,31]
Prognosis	[6,12,13,32,33,34]
Risk stratification	[10,35,36,37,38,39,40,41]
**Ultrasound**	
Cancer detection and diagnosis	[3,5,8,17,42,43,44,45,46]
Prognosis	[47,48]
Surgical planning	[15]
**MRI**	
Technique	[49,50]
Cancer detection	[51,52,53]
Cancer diagnosis—lesion characterization	[51,54]
Prognosis	[55,56,57,58,59,60,61,62,63,64,65,66,67,68,69,70,71,72]
Risk stratification	[11,73,74]
**Surgical planning**	[15,75,76,77,78,79]

**Table 2:** Artificial intelligence (AI) algorithms and their purposes.

AI Algorithm	Purpose	Techniques
**Mammography**		
Quality	Improve image acquisition by providing real-time feedback regarding position and quality control metrics and aggregate data to help establish trends between staff members	
Detection	Detect areas that need to be addressed by a radiologist	Computer-aided detection (CAD) AI, deep convolutional neural networks (CNN)
Prognostic factors	Automated estimate of fibroglandular tissue, which is correlated with breast cancer risk	
Risk stratification	Predicts the upgrade rate of in situ cancer to invasive malignancy and predicts 5 year risk of developing breast cancer	CNN and radiomics
**Ultrasound**		
Diagnosis	Provides decision support that ultimately improves accurate BI-RADS classification	CNN and an additional algorithm for classification
Prognostic factors	Ultrasound features, such as triple-negative breast cancer, used to predict the risk of recurrence	Radiomics analysis
Surgical planning	Assess vascular supply of the breast to determine the plausibility of reconstructive techniques	
**Magnetic Resonance Imaging (MRI)**		
Technique	Accelerated image acquisition by improving signal processing and reducing image noise	Artificially filling k-space
Diagnosis	Assess radiomic features extracted from contrast-enhanced T1-weighted and T2-weighted images	Machine-based learning
Prognostic factors	Quantitative assessment of background parenchymal enhancement, which is a possible risk factor for breast cancer	CNN
Risk stratification	Predicts the upgrade rate of in situ cancer to invasive malignancy	

## Data Availability

No new data were created or analyzed in this study. Data sharing is not applicable to this article.
